# Supplementary material for: mtFociCounter for automated single-cell mitochondrial nucleoid quantification and reproducible foci analysis
Source: Nucleic Acids Res. 2023 Oct 18;51(21):e107. doi: 10.1093/nar/gkad864 (PMC10681798; doi:10.1093/nar/gkad864)
Supplement: gkad864_Supplemental_Files [file gkad864_supplemental_files.zip › mtFociCounter_Supplementary_20230905.pdf]

mtFociCounter for automated single-cell mitochondrial nucleoid quantification and reproducible foci analysis

Timo Rey[1#], Luis Carlos Tábara[1], Julien Prudent[1], Michal Minczuk[1]

[1]Medical Research Council Mitochondrial Biology Unit, University of Cambridge, Cambridge CB2 0XY, UK

[#]Corresponding author: timorey@hotmail.com

Supplementary Material

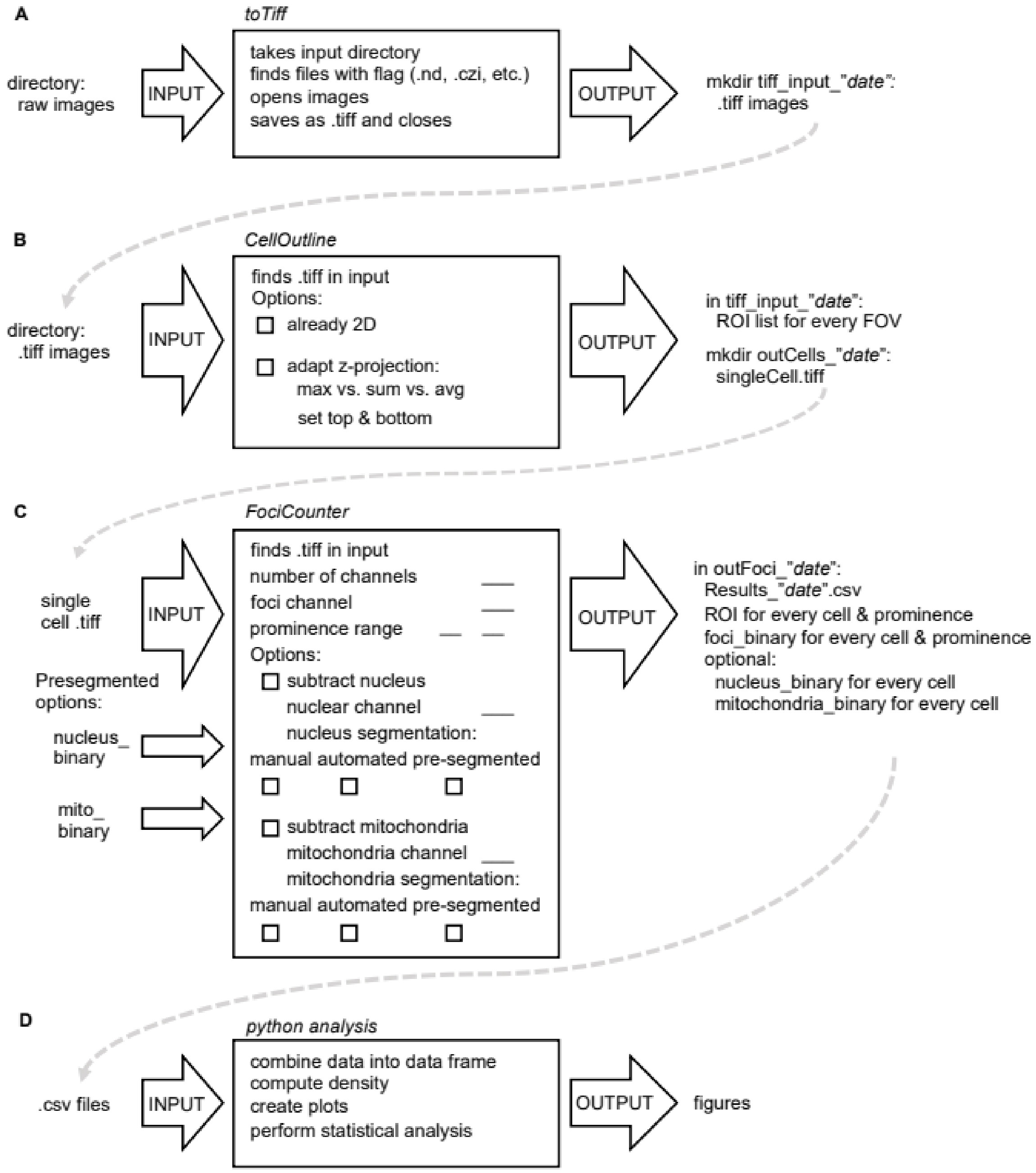

Supplementary Figure 1. Software-architecture of *mtFociCounter*.

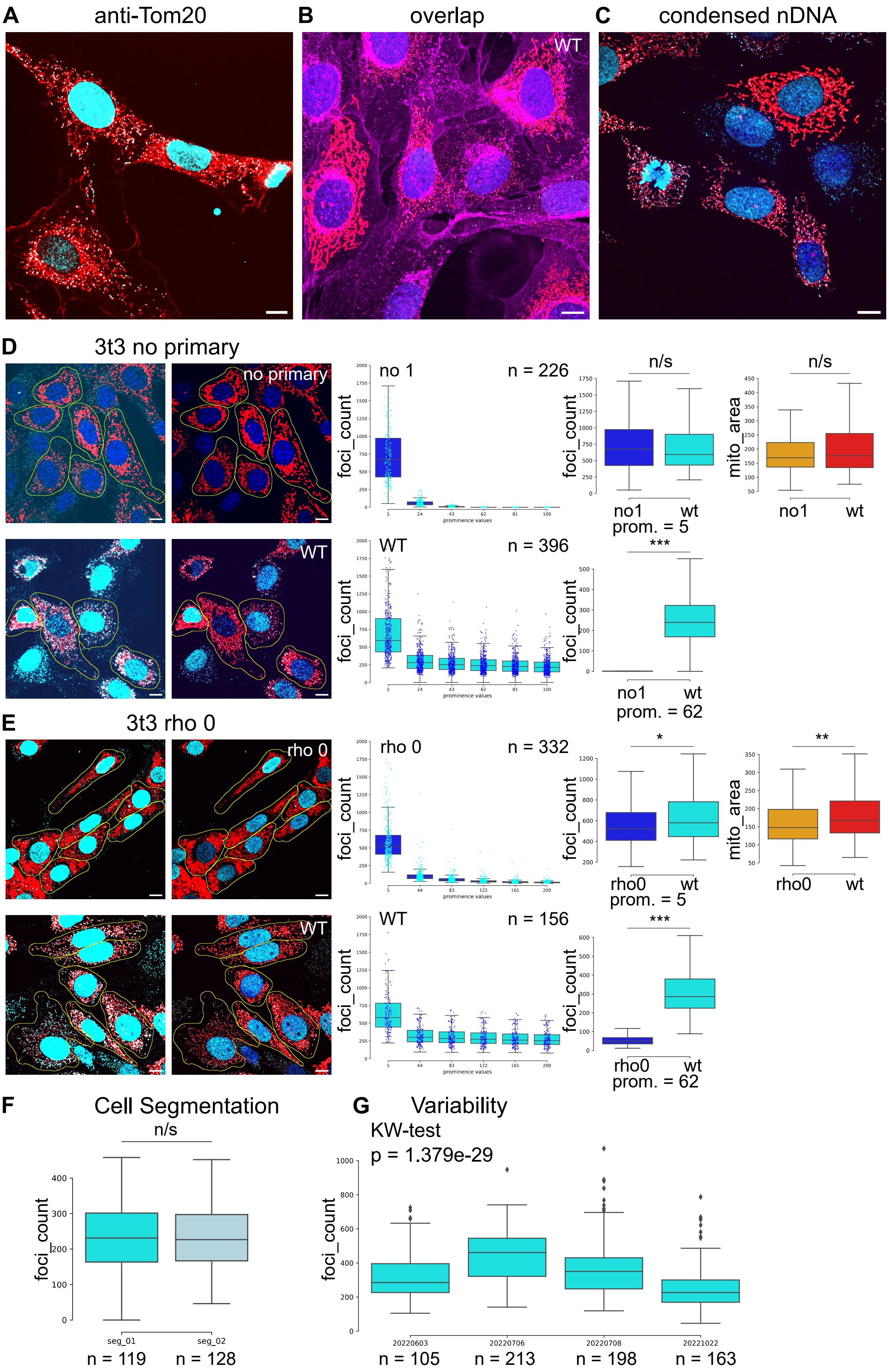
A
anti-Tom20
B
overlap
WT
C
condensed nDNA
D
3t3 no primary
no primary
no 1
n = 226
foci_count
prominence values
n/s
no1
wt
prom. = 5
mito_area
WT
n = 396
***
prom. = 62
E
3t3 rho 0
rho 0
n = 332
*
**
rho0
n = 156
F
Cell Segmentation
n/s
seg_01
seg_02
n = 119
n = 128
G
Variability
KW-test
p = 1.379e-29
20220603
20220706
20220708
20221022
n = 105
n = 213
n = 198
n = 163

Supplementary Figure 2. Experimental set-up and reproducibility analysis. (**A**) Confocal image of 3t3 fibroblasts with immunostaining against dsDNA (cyan) and against TOMM20 (mitochondria, red). (**B**) Confocal image of 3t3 fibroblasts with nuclear staining (Hoecst, blue), cell-outline (WGA-488, magenta) and mitochondria (MTS-dsRed, red). (**C**) Confocal image of 3t3 fibroblasts with immunostaining against dsDNA (cyan), MTS-dsRed (red) and Hoechst (blue) showing an actively dividing cell at the centre left. (**D**) Comparative analysis of 3t3 fibroblasts stained with or without a primary antibody. Left, four images from two representative FOVs from samples stained with (bottom row) or without (top row) primary antibodies. Contrast was manually but equally adjusted to highlight all foci based on "no primary" image (left column) or based on control image (right column). Centre, box and scatter plots of number of foci for each prominence value from a prominence sweep. Total number of cells analysed, from the combination of three independent experiments for each condition, are indicated. Right, box plots for direct comparison between primary-less (no1, blue and orange) and primary-stained (wt, cyan and red) of number of detected foci (left) for two prominence values, 5 or 62, and mitochondrial area. (**E**) As **D** for comparison between 3t3 rho0 cells, lacking mtDNA and 3t3 WT cells with normal mtDNA, both stained with antibodies against dsDNA (cyan). (**F**) Box plots for comparison of the number of nucleoids detected from the same sample after two separate rounds of cell segmentation (n=119 analysed cells after first segmentation and n=128 analysed cells after second segmentation). (**G**) Box plots of number of nucleoids detected in four independent experiments. Number of cells analysed for each experiment are indicated and p-value from Kruskal-Wallis test is indicated. All scale bars are 10μm. All box plots show median, first and third quartiles as well as the remaining distributions as whiskers. Two-sample Kolmogorov-Smirnoff tests were used for **D** to **F**, with alpha = 5% and * denoting $p<0.05$, ** denoting $p<0.001$, *** denoting $p<0.0001$.

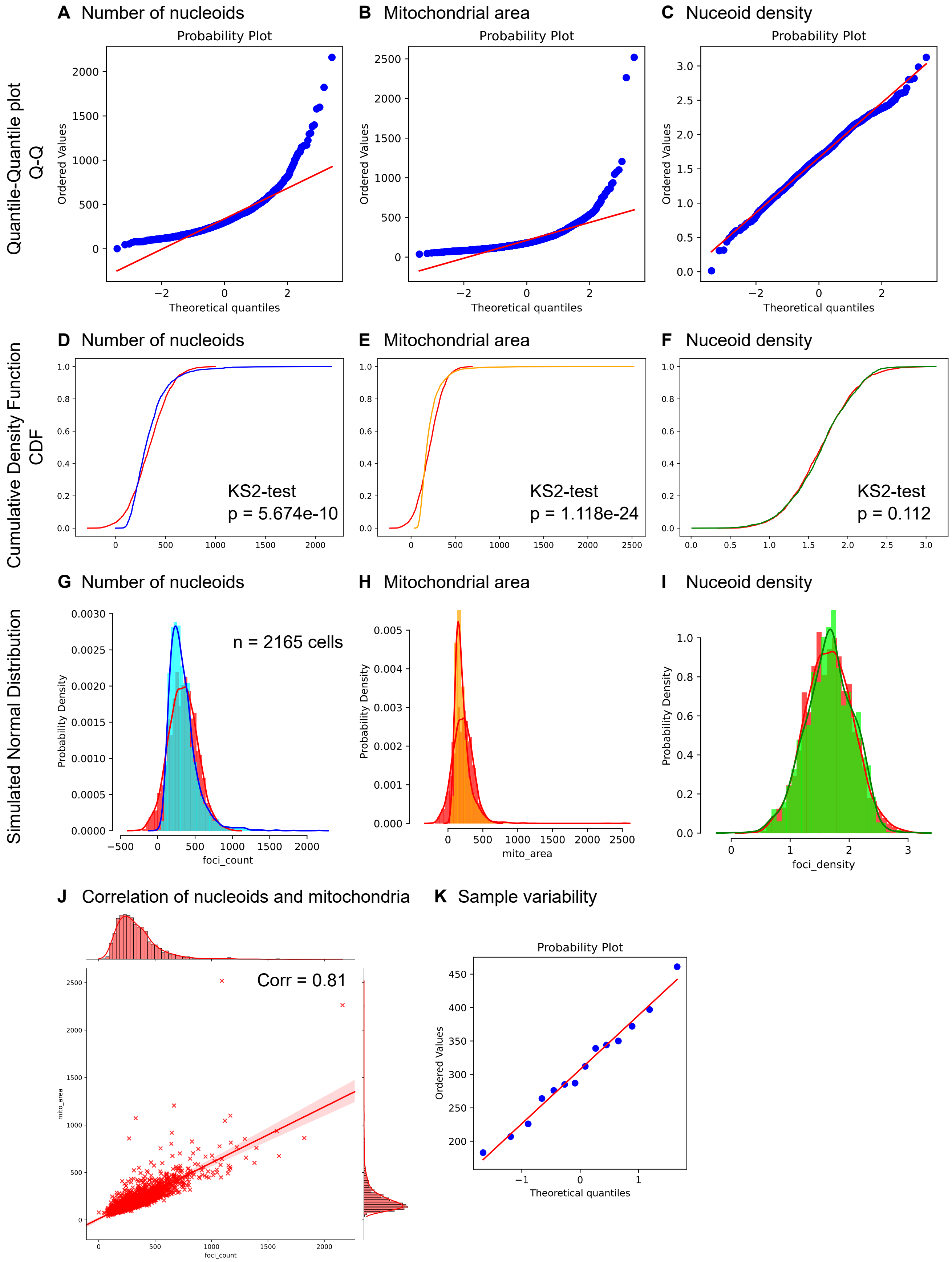
A Number of nucleoids
B Mitochondrial area
C Nuceoid density
Quantile-Quantile plot
Q-Q
Probability Plot
Ordered Values
Theoretical quantiles
D Number of nucleoids
E Mitochondrial area
F Nuceoid density
Cumulative Density Function
CDF
KS2-test
p = 5.674e-10
p = 1.118e-24
p = 0.112
G Number of nucleoids
H Mitochondrial area
I Nuceoid density
Simulated Normal Distribution
n = 2165 cells
Probability Density
foci_count
mito_area
foci_density
J Correlation of nucleoids and mitochondria
K Sample variability
Corr = 0.81

Supplementary Figure 3. **Distribution and correlative analysis of single-cell descriptors for 3t3 WT cells.** (**A**) Testing Normality of the data distribution by Q-Q plots for mitochondrial nucleoids, (**B**) mitochondrial area and (**C**) nucleoid density. (**D - F**) Comparison between the observed distribution and a simulated Normal distribution based on the observed mean, variance and sample size by 2-sample Kolmogorov-Smirnoff tests (p values are indicated) and visualisation of the respective Cumulative Density Functions, with simulations in red. Overlay of the observed distribution for the number nucleoids (**G**) (cyan), mitochondrial area (**H**) (orange) and nucleoid density (**I**) (green) and the simulated distributions (red). (**J**) Scatterplot, regression line and histogram with KDE plots for correlation analysis of mitochondrial area and the number of nucleoids detected in single cells. The correlation of 0.81 between mitochondrial nucleoid number and mitochondrial area is indicated. (**K**) Q-Q plot analysis of median number of nucleoids detected in 14 independent experiments. For all plots, the full data set of 2165 cells from 14 independent experiments, as described in **Figure 3**, was analysed.

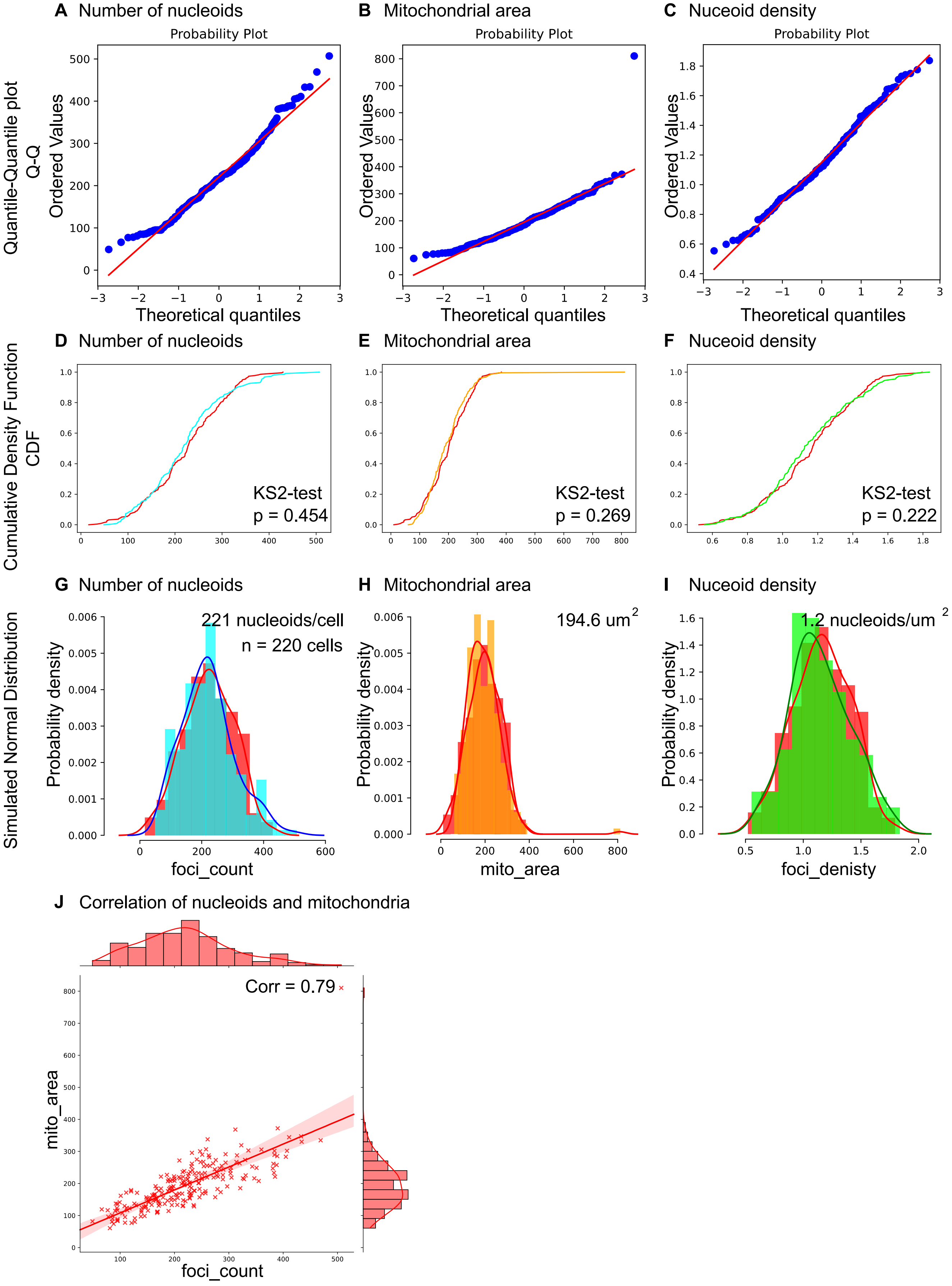
A Number of nucleoids
B Mitochondrial area
C Nuceoid density
Probability Plot
Quantile-Quantile plot Q-Q
Ordered Values
Theoretical quantiles
D Number of nucleoids
E Mitochondrial area
F Nuceoid density
Cumulative Density Function CDF
KS2-test
p = 0.454
KS2-test
p = 0.269
KS2-test
p = 0.222
G Number of nucleoids
H Mitochondrial area
I Nuceoid density
Simulated Normal Distribution
Probability density
221 nucleoids/cell
n = 220 cells
194.6 um 2
1.2 nucleoids/um 2
foci_count
mito_area
foci_denisty
J Correlation of nucleoids and mitochondria
Corr = 0.79
mito_area
foci_count

Supplementary Figure 4. Distribution and correlative analysis of single-cell descriptors for 220 U2OS cells treated with neutral siRNA. Analogous to **Supplementary Figure 3**. (**A**) Testing Normality of the data distribution by Q-Q plots for mitochondrial nucleoids, (**B**) mitochondrial area and (**C**) nucleoid density. (**D - F**) Comparison between the observed distribution and a simulated Normal distribution based on the observed mean, variance and sample size by 2-sample Kolmogorov-Smirnoff tests (p values are indicated) and visualisation of the respective Cumulative Density Functions, with simulations in red. Overlay of the observed distribution for the number nucleoids (**G**) (cyan), mitochondrial area (**H**) (orange) and nucleoid density (**I**) (green) and the simulated distributions (red). (**J**) Scatterplot, regression line and histogram with KDE plots for correlation analysis of mitochondrial area and the number of nucleoids detected in single cells. The correlation of 0.79 between mitochondrial nucleoid number and mitochondrial area is indicated. For all plots, the full data set of 220 cells from 3 independent experiments, as described in **Figure 4**, was analysed.

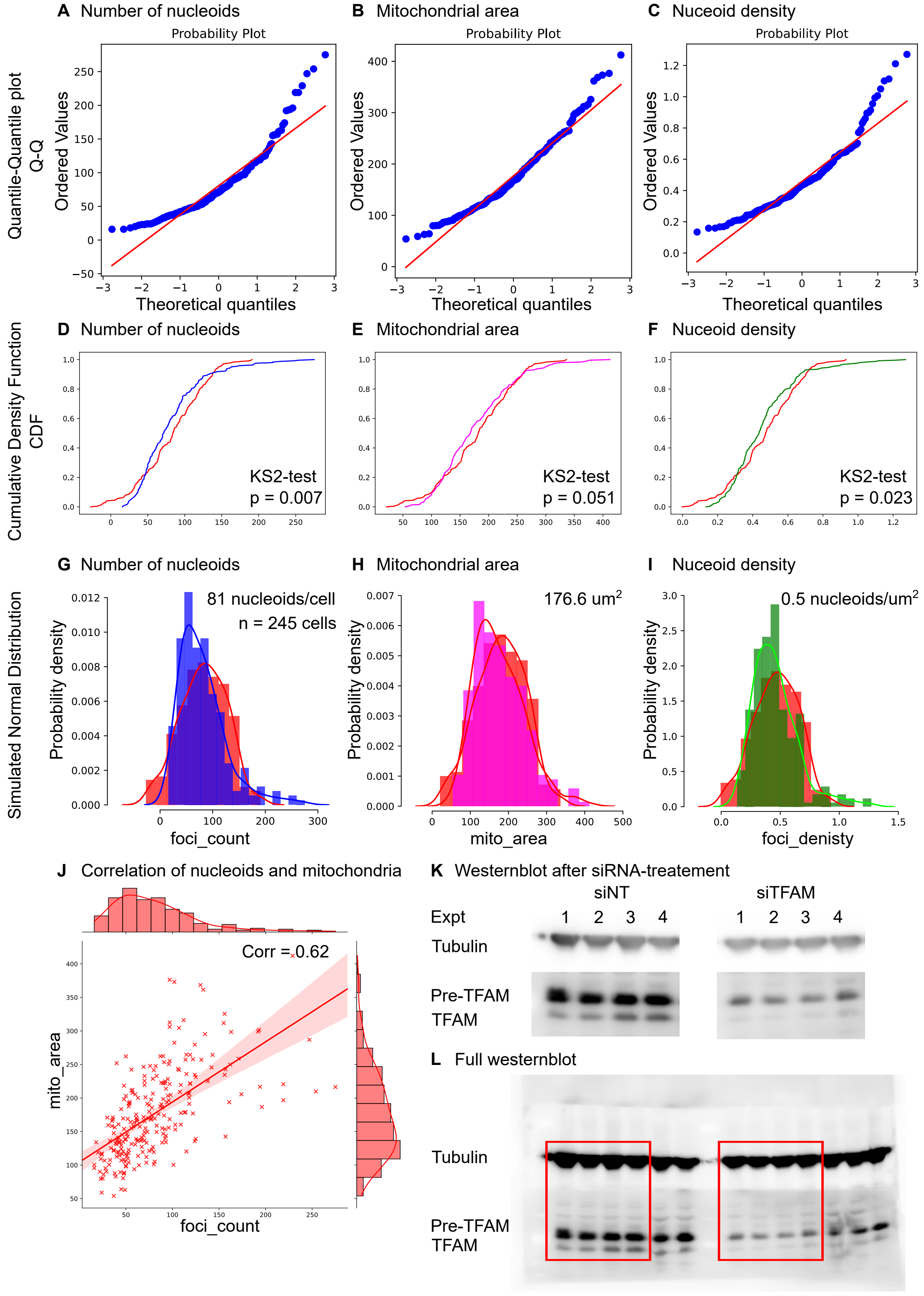
A Number of nucleoids
B Mitochondrial area
C Nuceoid density
Probability Plot
Quantile-Quantile plot
Q-Q
Ordered Values
Theoretical quantiles
D Number of nucleoids
E Mitochondrial area
F Nuceoid density
Cumulative Density Function
CDF
KS2-test
p = 0.007
KS2-test
p = 0.051
KS2-test
p = 0.023
G Number of nucleoids
H Mitochondrial area
I Nuceoid density
Simulated Normal Distribution
Probability density
81 nucleoids/cell
n = 245 cells
176.6 um2
0.5 nucleoids/um2
foci_count
mito_area
foci_denisty
J Correlation of nucleoids and mitochondria
Corr = 0.62
mito_area
foci_count
K Westernblot after siRNA-treatement
siNT
siTFAM
Expt
1 2 3 4
1 2 3 4
Tubulin
Pre-TFAM
TFAM
L Full westernblot
Tubulin
Pre-TFAM
TFAM

Supplementary Figure 5. Distribution and correlative analysis of single-cell descriptors for 245 U2OS cells treated with siTFAM. Analogous to **Supplementary Figure 3**. (**A**) Testing Normality of the data distribution by Q-Q plots for mitochondrial nucleoids, (**B**) mitochondrial area and (**C**) nucleoid density. (**D - F**) Comparison between the observed distribution and a simulated Normal distribution based on the observed mean, variance and sample size by 2-sample Kolmogorov-Smirnoff tests (p values are indicated) and visualisation of the respective Cumulative Density Functions, with simulations in red. Overlay of the observed distribution for the number nucleoids (**G**) (blue), mitochondrial area (**H**) (orange) and nucleoid density (**I**) (green) and the simulated distributions (red). (**J**) Scatterplot, regression line and histogram with KDE plots for correlation analysis of mitochondrial area and the number of nucleoids detected in single cells. The correlation of 0.62 between mitochondrial nucleoid number and mitochondrial area is indicated. For all plots, the full data set of 245 cells from 3 independent experiments, as described in **Figure 4**, was analysed. (**K**) Enlarged sections from Western Blot, as highlighted in red in full Western Blot in (**L**). Control staining against tubulin was used to monitor loading. Both, pre-TFAM and TFAM are visualised by anti-TFAM antibodies and siNT and siTFAM samples were processed in parallel. The four highlighted experimental samples correspond to the samples used for imaging experiments throughout the manuscript.

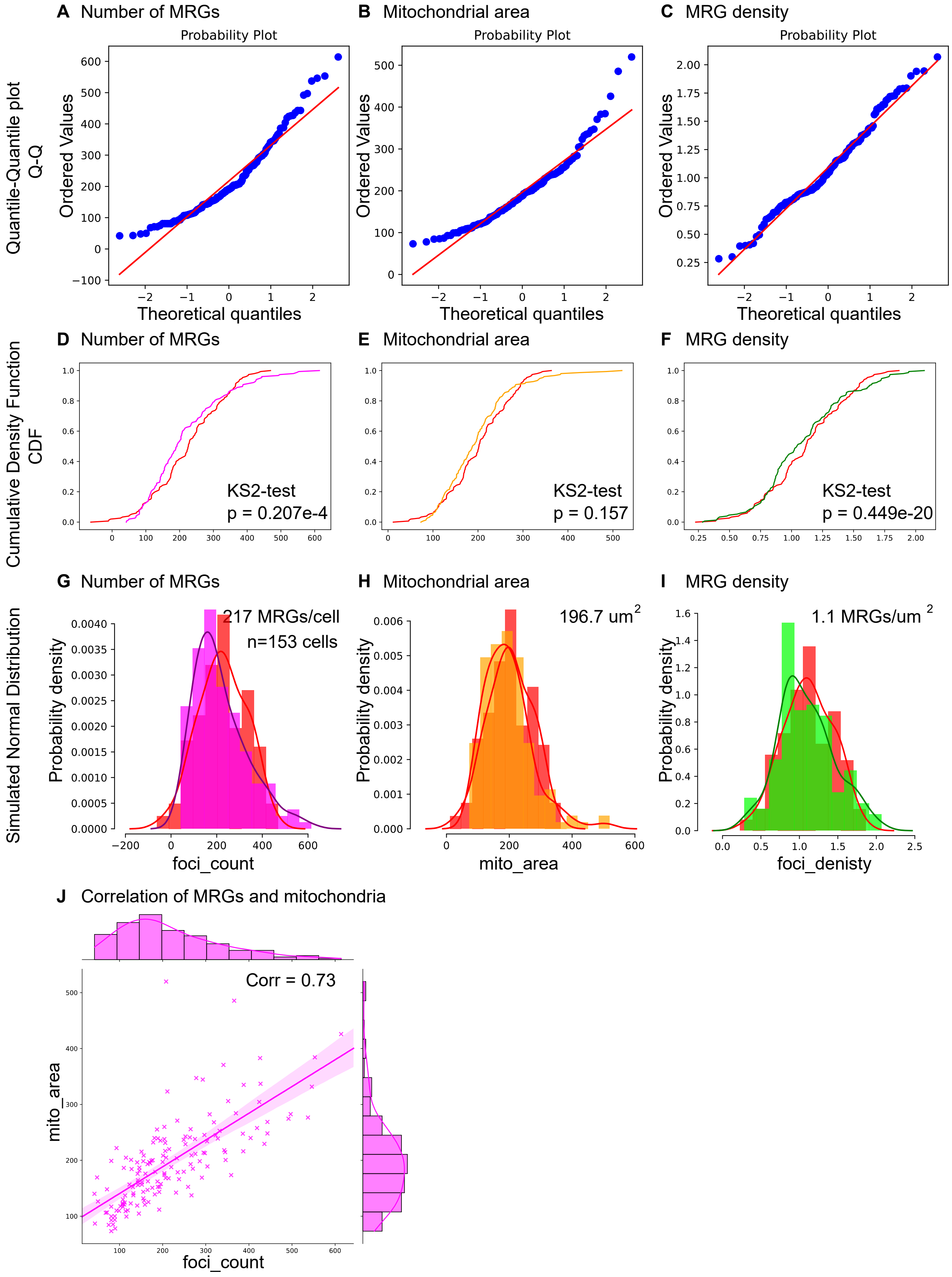
A Number of MRGs
B Mitochondrial area
C MRG density
Probability Plot
Quantile-Quantile plot Q-Q
Ordered Values
Theoretical quantiles
D Number of MRGs
E Mitochondrial area
F MRG density
Cumulative Density Function CDF
KS2-test
p = 0.207e-4
KS2-test
p = 0.157
KS2-test
p = 0.449e-20
G Number of MRGs
H Mitochondrial area
I MRG density
Simulated Normal Distribution
217 MRGs/cell
n=153 cells
196.7 um2
1.1 MRGs/um 2
Probability density
foci_count
mito_area
foci_denisty
J Correlation of MRGs and mitochondria
Corr = 0.73
mito_area
foci_count

Supplementary Figure 6 Distribution and correlative analysis of single-cell descriptors for MRGs in 153 single U2OS WT cells. (A) Testing Normality of the data distribution by Q-Q plots for MRGs, **(B)** mitochondrial area and **(C)** MRG density. **(D - F)** Comparison between the observed distribution and a simulated Normal distribution based on the observed mean, variance and sample size by 2-sample Kolmogorov-Smirnoff tests (p values are indicated) and visualisation of the respective Cumulative Density Functions, with simulations in red. Overlay of the observed distribution for the number MRGs **(G)** (magenta), mitochondrial area **(H)** (orange) and MRG density **(I)** (green) and the simulated distributions (red). **(J)** Scatterplot, regression line and histogram with KDE plots for correlation analysis of mitochondrial area and the number of MRGs detected in single cells. The correlation of 0.73 between MRG number and mitochondrial area is indicated. For all plots, the full data set of 153 cells from 3 independent experiments, as described in **Figure 4**, was analysed.

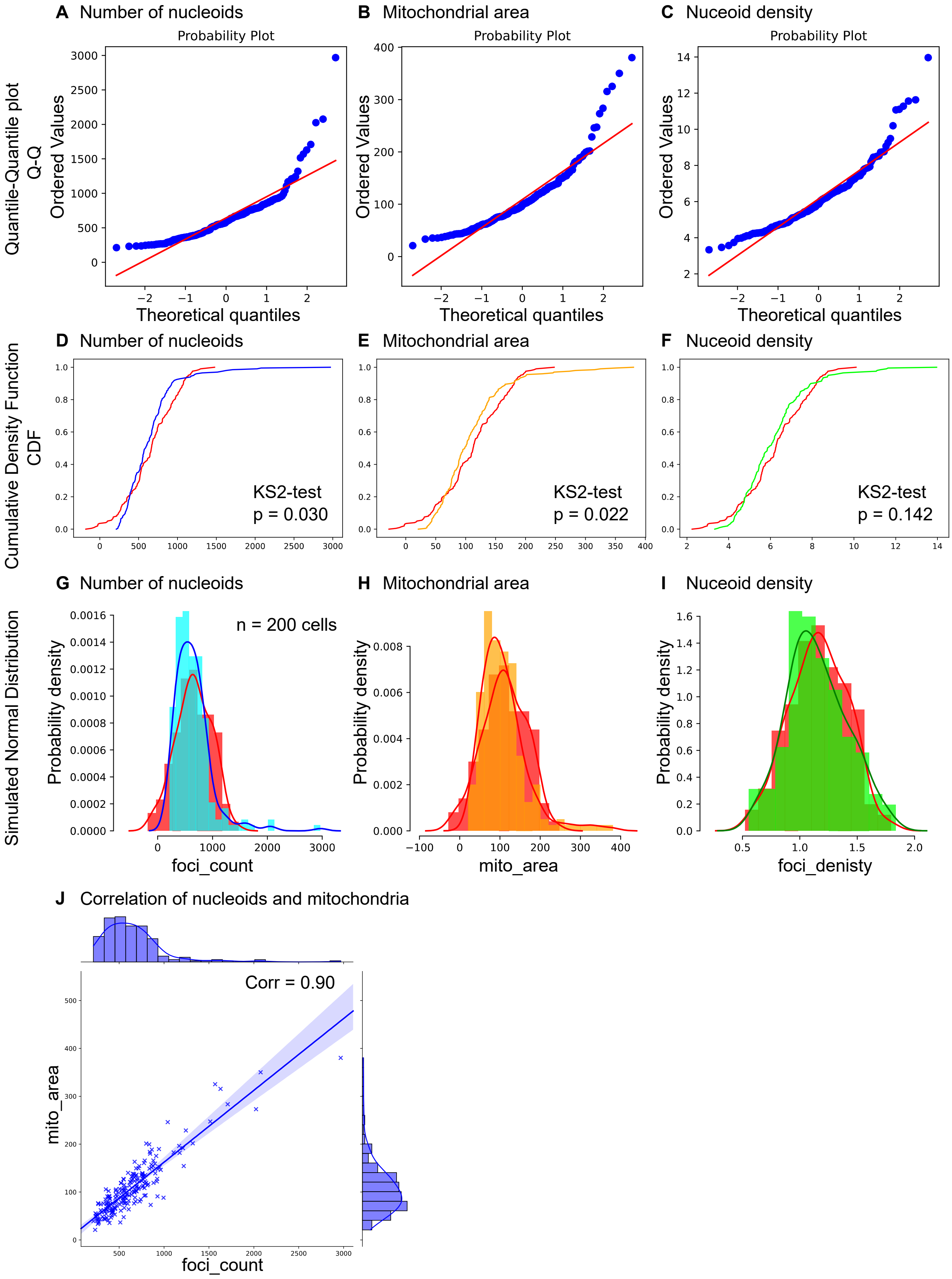
A Number of nucleoids
B Mitochondrial area
C Nuceoid density
Quantile-Quantile plot
Q-Q
Probability Plot
Ordered Values
Theoretical quantiles
D Number of nucleoids
E Mitochondrial area
F Nuceoid density
Cumulative Density Function
CDF
KS2-test
p = 0.030
KS2-test
p = 0.022
KS2-test
p = 0.142
G Number of nucleoids
H Mitochondrial area
I Nuceoid density
Simulated Normal Distribution
Probability density
n = 200 cells
foci_count
mito_area
foci_denisty
J Correlation of nucleoids and mitochondria
Corr = 0.90
mito_area
foci_count

Supplementary Figure 7 Distribution and correlative analysis of single-cell descriptors for 200 3t3 WT cells imaged by superresolution microscopy. Analogous to **Supplementary Figure 3**. (**A**) Testing Normality of the data distribution by Q-Q plots for mitochondrial nucleoids, (**B**) mitochondrial area and (**C**) nucleoid density. (**D - F**) Comparison between the observed distribution and a simulated Normal distribution based on the observed mean, variance and sample size by 2-sample Kolmogorov-Smirnoff tests (p values are indicated) and visualisation of the respective Cumulative Density Functions, with simulations in red. Overlay of the observed distribution for the number nucleoids (**G**) (blue), mitochondrial area (**H**) (orange) and nucleoid density (**I**) (green) and the simulated distributions (red). (**J**) Scatterplot, regression line and histogram with KDE plots for correlation analysis of mitochondrial area and the number of nucleoids detected in single cells. The correlation of 0.90 between mitochondrial nucleoid number and mitochondrial area is indicated. For all plots, the full data set of 200 cells from 3 independent experiments, as described in **Figure 5**, was analysed.

Data Availability

High resolution versions of supplementary figures and raw images are available on Zenodo: https://doi.org/10.5281/zenodo.7838928

All data and code are freely accessible on the data-repository Zenodo (DOIs: 10.5281/zenodo.7633157, 10.5281/zenodo.7634535, 10.5281/zenodo.7634645, 10.5281/zenodo.7634604) and Github (https://github.com/TimoHenry) respectively. Please do not hesitate to contact the authors in case of unclarity, questions about the use, suggestions for improvements or any other queries. Please make changes to the source code and submit improvements via Github. Biological material may be available upon reasonable request.
